# Accuracy and Safety of AI-Enabled Scribe Technology: Instrument Validation Study

**DOI:** 10.2196/64993

**Published:** 2025-01-27

**Authors:** Joshua Biro, Jessica L Handley, Nathan K Cobb, Varsha Kottamasu, Jeffrey Collins, Seth Krevat, Raj M Ratwani

**Affiliations:** 1 National Center for Human Factors in Healthcare MedStar Health Research Institute Washington, DC United States; 2 MedStar Health Institute for Innovation Washington, DC United States; 3 Georgetown University School of Medicine Washington, DC United States

**Keywords:** artificial intelligence, AI, patient safety, ambient digital scribe, AI-enabled scribe technology, AI scribe technology, scribe technology, accuracy, safety, ambient scribe, digital scribe, patient-clinician, patient-clinician communication, doctor-patient relationship, doctor-patient communication, patient engagement, patient safety, dialogue script, scribe

## Abstract

Artificial intelligence–enabled ambient digital scribes may have many potential benefits, yet results from our study indicate that there are errors that must be evaluated to mitigate safety risks.

## Introduction

Generative artificial intelligence (AI)–enabled ambient digital scribe (ADS) technology uses the patient-clinician conversation to generate clinical documentation; it has the potential to improve patient engagement and reduce clinician burden [1,2]. These technologies are becoming more prevalent, especially in ambulatory care settings, yet there is little known about documentation accuracy and the types of errors that may stem from ADS use [3]. Error-prone ADS technology may have serious patient safety consequences [4]. We evaluated 2 popular commercially available ADS products in a simulated setting to systematically identify the frequency and pattern of documentation errors.

## Methods

### Ethical Considerations

This study was approved by the MedStar Health Institutional Review Board (00007789) to cover secondary analysis of existing patient data without additional consent. All data were deidentified. Participants did not receive any form of compensation.

### Recording and Simulation

Recordings of 11 real outpatient encounters from a range of service lines (otolaryngology, cardiology, rheumatology, family medicine, pediatrics, endocrinology, internal medicine, gastroenterology, oncology, and urgent care) were transcribed by automated software and then deidentified and edited by a senior physician (NKC) for clarity to create 11 unique dialogue scripts. The dialogue scripts were used to evaluate 2 commercial ADS products. For each script, a researcher (JB or VK) simulating the patient and a medical resident simulating the physician read from the script while the ADS products were in use. Each script was read by 2 different residents per ADS product, yielding 22 draft notes per product and 44 draft notes in total across products. The residents reviewed the draft notes to identify errors. Each error was independently categorized by 2 reviewers (JB or JLH) as either an omission, addition, wrong output, or irrelevant or misplaced text, as defined in Table 1. Disagreements were discussed to reach consensus.

## Results

There were 127 errors (mean 2.9, SD 2.7 errors per draft note) in 31 of 44 (70%) draft notes. ADS product A resulted in 66 errors (mean 3, SD 2.7 per draft note) and product B resulted in 61 errors (mean 2.8, SD 2.7 per draft note). Error frequency by error type and product is detailed in Table 1, with omission errors being the most frequent across products. Error types significantly differed between the 2 ADS products (Fisher exact test: *P*=.002).

**Table 1:** Frequency counts, percentages, definitions, and examples of ambient digital scribe (ADS) error types.

Error type	Errors by ADS product, n (%)		Definition	Example
	Product A (n=66)	Product B (n=61)		
Omission	55 (83)	33 (54)	Model leaves out key information from its response	“[N]o laterality mentioned in ears in physical exam section”
Addition	3 (4)	7 (11)	Model adds inappropriate or irrelevant information	“Patient doesn’t refer to any flare ups in awhile, but the note shared that patient was using X medication to help with flare ups in HPI”
Wrong output	4 (6)	6 (10)	Model provides an incorrect response	“[A]ssociated the wrong test with the contrast”
Irrelevant or misplaced text	4 (6)	15 (25)	Model output is technically correct but not appropriate in clinical context	“[C]aptured all the supplemental information (asthma, mammogram, etc.) and harped on the steroid injections which doesn’t matter”

## Discussion

While ADS technologies may have potential benefits, there are frequent errors in the generated note. Across both products, errors of omission were the most common; this error type may be the most difficult for clinicians to identify since the identification process requires memory recall of details from the patient encounter. If clinicians review their documentation after several patient encounters, recalling omitted details may be challenging. It may be easier to identify errors such as additions and wrong outputs since this relies on recognition of an issue in the text being presented to the clinician. Notably, there was a different pattern of errors between the two products.

There are limitations to this study. The ADS technologies were evaluated against a limited number of patient cases in a controlled environment that did not fully represent clinical workflows. In addition, the cases were read by 2 researchers acting as patients, and it is likely that both clinicians and patients would have more variability in language, tone, volume, and many other characteristics that could impact ADS accuracy.

It is imperative that ADS technologies be evaluated in a realistic clinical setting (either in situ or in a representative simulation) to determine the frequency and types of errors so that appropriate risk mitigation and safety plans can be developed. Developing methods to capture AI-related safety issues was a component of President Joe Biden’s “Executive Order on the Safe, Secure, and Trustworthy Development and Use of Artificial Intelligence” [5], and robust processes for AI safety are needed. In the absence of a standardized evaluation framework, health care facilities currently bear the burden of testing and reporting these results in the United States. It is to be noted that, effective August 2024, the European Union Artificial Intelligence Act legally requires developers of AI-based systems to evaluate the safety of their products [6]. While a step in the right direction, the underlying vendor algorithms are often proprietary, opaque, and the subject of continuous innovation; thus, there is still a need for independent ongoing testing to confirm vendor claims of safety. Future work should develop a robust, standardized, and repeatable ADS evaluation framework to facilitate efficient knowledge sharing in this fast-paced, decentralized system.

## Abbreviations

ADS: ambient digital scribe
AI: artificial intelligence

## Data Availability

The data sets generated during and/or analyzed during this study are available from the corresponding author on reasonable request.
